# The long-term efficacy of transoral incisionless fundoplication with Medigus Ultrasonic Surgical Endostapler (MUSE) for gastroesophageal reflux disease

**DOI:** 10.1007/s10388-023-00992-3

**Published:** 2023-03-06

**Authors:** Shien Shen, Ge Yu, Xingya Guo, Guanzhao Zong, Chuanyang Wang, Jingpiao Bao, Jiahui Chen, Zhiyuan Cheng, Wenqin Xiao, Jie Shen, Weiliang Jiang, Rong Wan

**Affiliations:** 1 Department of Gastroenterology, Shanghai General Hospital, Shanghai Jiao Tong University School of Medicine, 85 Wujin Road, Shanghai, 200080 China; 2 Shanghai General Hospital of Nanjing Medical University, Nanjing, China

**Keywords:** Gastroesophageal reflux, Medigus Ultrasonic Surgical Endostapler (MUSE), Follow-up, Transoral incisionless fundoplication, Proton pump inhibitor

## Abstract

**Background:**

To evaluate the long-term efficacy of transoral incisionless fundoplication (TIF) with Medigus Ultrasonic Surgical Endostapler (MUSE) for gastroesophageal reflux disease (GERD).

**Methods:**

A total of 16 patients with proton pump inhibitor-dependent gastroesophageal reflux disease had undergone TIF by MUSE in Shanghai General Hospital （Shanghai, China）from March 2017 to December 2018. Patients were followed up at 6 months, and the GERD-health-related quality of life (GERD-HRQL) questionnaire score, the GERD questionnaire (GERD-Q) score, high-resolution esophageal manometry (HREM) and 24 h esophageal pH parameters, the Hill grade of the gastroesophageal flap valve (GEFV) and daily Proton pump inhibitor (PPI) consumption before and after procedure were compared. Patients also were followed up at 3 years and 5 years using a structured questionnaire via phone which evaluated symptoms of reflux, dose of PPI medication and side effects.

**Results:**

Follow-up data were collected from 13 patients, ranging from 38 to 63 months, 53 months on average. 10/13 patients reported symptomatic improvement and daily PPI consumption was stopped or halved in 11/13. After procedure, the mean scores of GERD-HRQL and GERD-Q were significantly increased. The mean DeMeester score, the mean acid exposure time percentage and the mean number of acid reflux episodes were significantly lower. The mean rest pressure at lower esophageal sphincter (LES) had no significant difference.

**Conclusion:**

TIF by MUSE has significant efficacy in the treatment of PPI-dependent GERD, which can improve symptoms and life quality of patients, and reduce the acid exposure time for long-term. Chictr.org.cn.

**Trial Registration:**

ChiCTR2000034350.

## Introduction

Gastroesophageal reflux disease (GERD) is a common digestive disease characterized by acid reflux and heartburn [1]. In China, the change of diet structure makes the prevalence rate increase year by year with the development of society [2]. The high prevalence rate not only reduces the quality of life of the patients, but also brings a certain burden to the social economy [3]. The first choice for treatment of GERD is Proton pump inhibitor (PPI), still many patients relapse after treatment and need to take PPI for a long time to control their symptoms. However, prolonged use of PPI increases risk of infection, reduced intestinal absorption of vitamins and minerals, and more recently kidney damage and dementia [4]. Guidelines of GERD recommend anti-reflux surgery for patients who do not want to take long-term PPI therapy [5]. Transoral incisionless fundoplication (TIF) has proved an effective therapeutic option which is safer than surgical fundoplication and laparoscopic fundoplication [6, 7]. Medigus Ultrasonic Surgical Endostapler (MUSE) is a new device for TIF, which is proved by Food and Drug Administration (FDA). TIF by MUSE based on the anti-reflux function of the esophageal valve mechanism, enforces the gastric fundus to the esophageal wall 3 cm above the gastroesophageal junction from different angles via endoscopy, thereby strengthening the anti-reflux valve flap and restoring the His angle, to establish effective barriers to prevent acid reflux disease and achieve the goal of anti-reflux. In order to evaluate the short-term and long-term effects of TIF by MUSE and to observe whether there are any postoperative complications, a retrospective analysis of GERD cases treated with Muse in our hospital several years ago was conducted, to evaluate the safety of the operation.

## Methods

Between March 2017 and December 2018, sixteen men and women with proton pump inhibitor-dependent gastroesophageal reflux disease were enrolled in the study.

Inclusion criteria were as follows: (a) age > 18 and < 70 years; (b) chronic (at least 1 year) GERD-related esophageal symptoms, response to PPI therapy, but need long-term treatment (at least 6 months); (c) pH < 4 more than 4.2% of time; (d) esophageal peristalsis is normal; (e) ability to read and sign Intensive care facility (ICF).

Exclusion criteria were as follows: (a) hiatal hernia ≥ 2 cm; (b) Barrett’s esophagus; (c) Los Angeles grade D esophagitis; (d) esophagus or gastric varices; (e) previous GI or thoracic surgery; (f) gastroesophageal valve Hill grade I or grade IV; (g) achalasia of cardia; (h) malignant upper GI neoplasia; (i) serious systemic disease such as scleroderma; (j) body mass index > 35 kg/m^2^ or < 20 kg/m^2^; (k) inability to give consent and unavailability to long-term follow-up.

Before the intervention and 7 days after stopping PPIs, all patients completed the GERD-Health Related Quality of Life (GERD-HRQL) [8] and GERD questionnaire (GERD-Q) [9], and daily PPI consumption was assessed, PPI used by patients includes omeprazole, rabeprazole, pantoprazole and lansoprazole. Patients also underwent high-resolution esophageal manometry (HRM), 24-h ambulatory pH-impedance monitoring (always off PPI), and upper GI endoscopy. Six months after the procedure, the patients were re-examined and underwent repeat HRM, ambulatory acid exposure test, endoscopy, GERD-HRQL, GERD-Q, and were questioned regarding adverse events and medication use. Three and five years after the procedure, patients were subsequently interviewed by phone. The interview consisted of repeating the GERD-HRQL questionnaire, adverse events and medication use. Post-TIF daily PPI consumption was considered “unchanged,” “halved,” and “stopped”, when the daily drug dose was the same as before, half that used before, or when no PPIs were being taken during the follow-up.

### MUSE procedure

**Fig. 1:**
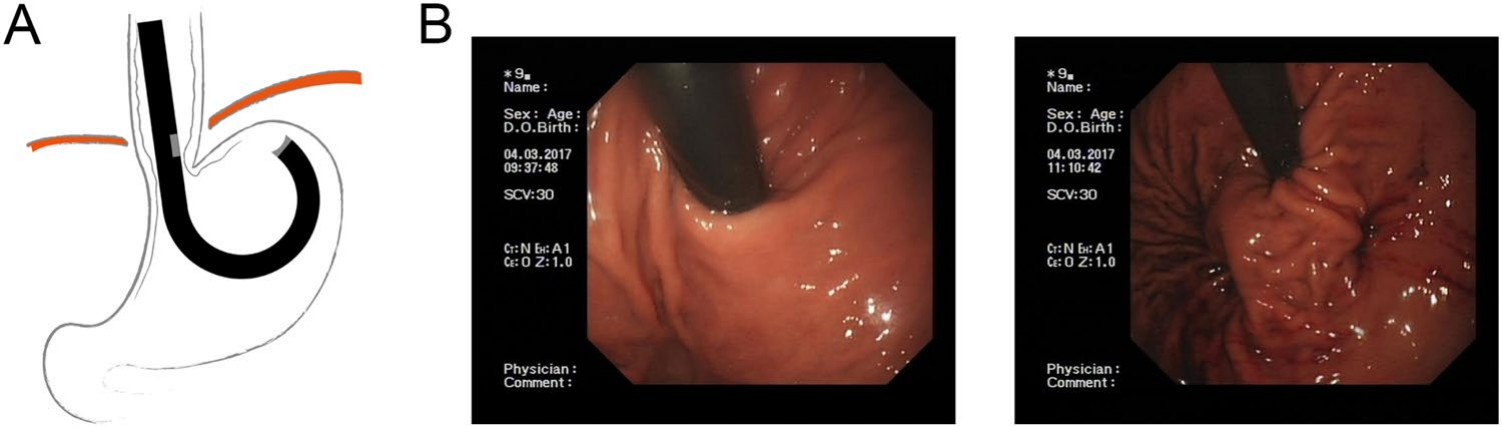
**A** MUSE procedure: the device was retroflexed in the fundus, and placed the cartridge 3 cm proximal to gastroesophageal junction for stapling; **B** the endoscopic images before and after TIF by MUSE

The device consists of endostapler and a console, including a camera and ultrasonic range finder, various sensors, irrigation and suction system. The endostapler has a handle with a controller, an insertion tube of 15.5 mm in diameter comprising operative channels, a cartridge containing five 4.8 mm titanium staples, the ultrasound mirror, electrical and mechanical cables that operate the device, one alignment pin funnel, and two anvil screw funnels. The distal tip can align with the cartridge (Fig. 1).

The procedures were performed under general anesthesia with endotracheal intubation in a therapeutic endoscopy suite. The device was introduced through the overtube, and retroflexed in the fundus, then pulled back to place the staple cartridge in the esophagus nearly 3 cm proximal to the gastroesophageal junction. When the tissues were compressed, the ultrasonic range finder automatically engaged to measure the tissue thickness. The operator delivered at least three quintuplets of staples when the tissue thickness was 1.4–1.6 mm. Antiemetic prophylaxis and muscle relaxation throughout the procedure were necessary. The operators of MUSE were all endoscopists who have more than 10 years of experience in endoscopic operation, and for the sake of safety, all operators had undergone theoretical training and animal surgery before the operation.

All patients were kept in hospital at least 2 days after the procedure. Broad-spectrum antibiotics and antiemetic prophylaxis were given intravenously for 48 h. Analgesic prophylaxis was routinely given for the first 24 h. If the patient experienced intense pain requiring major analgesics or high fever persisting for 2 days, a hydrosoluble contrast radiography and computed tomography (CT) scan were completed immediately (to exclude possible esophageal or gastric leakage). At discharge, patients were instructed to follow a liquid diet for 3 days and then a soft diet for the first weeks. PPIs were discontinued 2 weeks after the procedure. The study protocol was approved by the ethics committee of Shanghai General Hospital (N.2016-09), and registered at Chictr.org.cn. (Identifier: ChiCTR2000034350).

### Statistical analysis

All continuous variables are reported as median values with estimated 95% confidence intervals (CI), or as mean values ± standard deviation, based on test for normal distribution. Statistical significance was assessed through separate paired samples two-tailed Wilcoxon signed-rank sum test. Thresholds of statistical significance were set at 0.01 (alpha of 0.05/5). *P* values below thresholds were considered statistically significant.

## Results

TIF by MUSE was technically successful in 16/16 patients, with a mean (SD) procedure duration of 62 (24) minutes. After TIF, subcutaneous emphysema occurred in one (6.25%) patient, it was the only adverse event. 13 patients completed the all 5-year follow-up. Demographic data for the treated patients are provided in Table 1.

**Table 1:** Demographic and clinical and endoscopic data of the 13 patients

Demographic features	
Gender, *N*. (%)	
Male	11 (84.6)
Female	2 (15.4)
Age (years), mean ± SD	48.9 ± 11.1
BMI (kg/m^2^), mean ± SD	24.0 ± 3.8
Clinical features	
Duration of symptoms (years), mean ± SD	5.8 ± 4.4
Daily PPI consumption	
Double standard, *N*. (%)	7 (53.8)
Single standard, *N*. (%)	6 (46.2)
Endoscopic features	
Normal esophagus, *N*. (%)	3 (23.1)
Grade A esophagitis, *N*. (%)	6 (46.1)
Grade B esophagitis, *N*. (%)	3 (23.1)
Grade C esophagitis, *N*. (%)	1 (7.7)
Grade D esophagitis, *N*. (%)	0 (0.0)
Gastroesophageal valve Hill grade II, *N*. (%)	4 (30.8)
Gastroesophageal valve Hill grade III, *N*. (%)	9 (69.2)
No hiatal hernia	11 (84.6)
Hiatal hernia < 2 cm	2 (15.4)
Hiatal hernia ≥ 2 cm	0 (0.0)

*N* number, *SD* standard deviation, *BMI* body mass index, *PPI* proton pump inhibitor

The GERD-HRQL scores off therapy and daily PPI consumption at baseline, 6 months, 3 years and 5 years are summarized in Table 2. The primary success criterion was at least 50% improvement in GERD-HRQL scores. The mean GERD-HRQL was reduced from 26.5 at baseline to 3.6 at 6 months, and stable at around 7.1 at 5 years. 10/13 patients (76.9%) had improved GERD-HRQL scores by more than 50% at 3 and 5 years. Use of daily PPI was eliminated in 12/13 patients (92.3%), with 11/13 (84.6%) completely off of any PPIs, but at 3 years, some patients had increased PPI dose. Finally, 11/13 patients (84.6%) had eliminated PPI use ≥ 50% at 5 years. The GERD-Q scores off PPI were reduced from 14.6 at baseline to 7.4 at months.

**Table 2:** Results for GERD-HRQL and daily PPI consumption

Follow-up	Baseline	6 months	3 years	5 years
GERD-HRQL (off PPI)				
Total score, mean ± SD	26.5 ± 6.2	3.6 ± 61 *	8.9 ± 11.4*	7.1 ± 8.7*
GERD-HRQL score reduce > 50%, *N*. (%)	/	12 (92.3)	10 (76.9)	10 (76.9)
Daily PPI consumption				
Unchanged, *N*. (%)	/	1 (7.7)	3 (23.1)	2 (15.4)
Halved, *N*. (%)	/	1 (7.7)	4 (30.8)	6 (46.2)
Stopped, *N*. (%)	/	11 (84.6)	6 (46.1)	5 (38.4)

*Wilcoxon Signed-Rank test versus baseline value, *P* < 0.001

13/16 (81.3%) patients completed the 6 months scheduled endoscopic and functional investigation follow-up, and detailed data are reported in Table 3. The follow-up upper GI endoscopy showed that there were 5 cases of grade A esophagitis and 1 case of grade C esophagitis. The grade of esophagitis was reduced in 10 patients, no change in 3 patients and no exacerbation of esophagitis. At the procedure, the Hill grade of the newly created valve was I in all cases. At 6 months after the intervention, Hill grade of the gastroesophageal valve was I in 11/13 (84.6%) patients, and II in 2/13 (15.4%) patients. At 24-h impedance recording, percent total time pH ≤ 4, total number of refluxes and DeMeester score were significantly reduced. But at HRM, the median lower esophageal sphincter (LES) basal pressure had no significant difference compared to before treatment.

**Table 3:** Results for upper GI endoscopy and functional findings

Follow-up	Baseline	6 months	*P* value*
Upper GI endoscopy			
Gastroesophageal valve Hill grade(95% CI)	2.7 (2–3)	1.2 (1–2)	< 0.001
24-h pH-impedance			
Total time (%) esophageal pH ≤ 4, (95% CI)	10.5 (5.2–74.5)	3.3 (0.2–24.5)	0.005
Total number of refluxes (95% CI)	125 (62–336)	46.5 (9–207)	< 0.001
DeMeester score (95% CI)	35.7 (20.2–255.3)	11.9 (1.4–97.3)	0.006
HRM			
LES basal pressure (mmHg), mean ± SD	16.6 ± 10.1	15.4 ± 9.0	0.77

*CI* confidence interval, *HRM* high-resolution esophageal manometry, *LES* lower esophageal sphincter

*Comparisons of median values (95% CI) within parameters (Wilcoxon test; adjusted *P* value = 0.05/5 = 0.01)

## Discussion

PPI therapy and laparoscopic fundoplication have been the mainstay of GERD treatment. But their limitations and adverse effects have led to the development of alternative therapies including MUSE™, Stretta™, GERDx™, EsophyX™ and others [10–14]. Several clinical studies demonstrated the efficacy of TIF by MUSE. In Europe, a multi-center prospective study showed that the TIF by MUSE was relatively safe and efficacious with 4 years of follow-up for patients with PPI-responsive GERD [15]. Post-procedure, 31/37 (83.8%) and 25/36(69.4%) patients remained off daily PPI at 6 months and 4 years, respectively. The mean GERD-HRQL scores (off PPI) improved from 29.1 to 8.9 at 6 months, and 5.3 at 4 years post-procedure. Two severe adverse events requiring intervention occurred in the first 24 operations [16], and no new Serious Adverse Event (SAE) was reported after 6 months of follow-up. In another study, PPI consumption was stopped and halved in 38/42(90.5%), 31/35(88.6%), and 27/31(81.7%) of cases at 1, 2, and 3 years post-procedure, respectively [17]. GERD-HRQL decreased at least 50%, respectively, in 30/42 (71.5%), 25/35 (71.4%), and 21/31 (67.7%) of cases. Perforation occurred in two cases, and one patient required surgery within 6 months.

In our prospective study, the mean GERD-HRQL scores (off PPI) improved from 26.5 to 3.6 at 6 months, and stable at around 7.1 at 5 years. Ten-year follow-up data for Stretta procedure showed significant decrease in GERD-HRQL scores (baseline 27.81 to 8.55 at 10-year follow-up) [18]. Although only 5-year follow-up data were available in the current study, decrease in the mean GERD-HRQL score (off PPI, 29.1 to 8.5 at 10 years) showed similar results. GERD-HRQL scores reduced at least 50% in 12/13 (92.3%) and 10/13 (76.9%) of cases at 6 months and 5 years post-procedure, respectively. Daily PPI consumption was stopped and halved in 12/13 (92.3%), 10/13 (76.9%), 11/13 (84.6%) at 6 months, 3 and 5 years. When combined with the other two studies, we found a consistent trend toward reduced PPI consumption and decreased GERD-HRQL scores. The number of patients with improved symptoms was the highest at 6 months, and individual patients relapsed at 3 and 5 years. Subcutaneous emphysema occurred in one patient after TIF. One patient had chest discomfort but no difficulty swallowing at 3-year follow-up. Although we did not have a control group, the patient’s symptoms improved after 5 years, suggesting it was not placebo. Overall, TIF by MUSE was efficacious and safe for patients with PPI-dependent GERD.

The results for upper GI endoscopy and functional findings were similar to the results for GERD-HRQL and daily PPI consumption. Morphological assessment indicated that the Hill grade of the newly created valve remained I in 11/13 (84.6%) patients at 6-month endoscopy, and all others were II. Meanwhile, the esophagitis was improved in 10 patients, no change in 3 patients and no exacerbation of esophagitis. TIF by MUSE was able to create an long-term effective new valve. But in the functional parameters, the median lower esophageal sphincter (LES) basal pressure had no significant difference between baseline and 6 months. Possible reasons are the small number of patients or the degree of cooperation when the examination. At 24-h impedance recording, percent total time pH ≤ 4, total number of refluxes and DeMeester score were significantly reduced. This means that the patient’s acid exposure time was significantly reduced.

Through the analysis of ineffective cases, we found that the three cases had some similar characteristics. First, all three patients’ body mass index were more than 26 kg/m^2^, although effective cases also include patients with body mass index of more than 26 kg/m^2^. Obesity may be a risk factor for bad responder. Second, three patients had good symptom control at 6 months after surgery, but as time went on, the effect became worse and worse, and eventually relapsed. The endoscopic findings and symptoms were similar, the Hill grade of valve improved at 6 months after surgery, but returned to preoperative levels eventually. More research is needed to find out how to keep the operation effective in the long term.

In this protocol study, TIF was proposed to patients with Hill grade II or III of the valve or small hiatal hernias. The Hill grade IV was an exclusion criterion for MUSE therapy. Laparoscopic fundoplication with hiatal hernia repair was a better choice for the patients with medium to large-sized sliding hernia. Currently, the MUSE system is more expensive than laparoscopic fundoplication, but with technology advances, the price will gradually fall in the future. Although the operation of MUSE is not complicated, all operators should be trained and evaluated by Dr. Amir Govrin’s team, and those who pass the assessment will have qualifications. We strongly recommend that MUSE procedure should not be performed by operators who have never received training and had no qualification.

In conclusion, our prospective study reports the long-term follow-up results of safety and therapeutic effectiveness of TIF by MUSE in patients with PPI-dependent GERD. TIF by MUSE can improve GERD-HRQL scores as well as reducing PPI use in patients with GERD. Future studies should focus on the patients with longer term follow-up results of intraesophageal pH monitoring and the patients with poor PPI responsiveness.
